# Protecting Educators: A Scoping Review of Interventions That Address Teacher Victimization

**DOI:** 10.3390/bs15020214

**Published:** 2025-02-14

**Authors:** Sarah M. Stilwell, Paulina Guzmán, Jorge Varela, Susan D. McMahon, Kailyn Bare, Justin Heinze, Marc Zimmerman

**Affiliations:** 1 Department of Health Behavior and Health Equity, University of Michigan, Ann Arbor, MI 48105, USA; jheinze@umich.edu (J.H.); marcz@umich.edu (M.Z.); 2 Faculty of Education, San Sebastián University, Santiago 8420524, Chile; pauguzmanm@udd.cl; 3 Faculty of Psychology, University of Desarrollo, Santiago 7610658, Chile; jovarela@udd.cl; 4 Department of Psychology, DePaul University, Chicago, IL 60614, USA; smcmahon@depaul.edu (S.D.M.); kbare1@depaul.edu (K.B.)

**Keywords:** teacher victimization, scoping review, teacher-directed violence, interventions

## Abstract

The prevalence of teacher-directed violence has been well-documented around the world. However, interventions focused on the prevention of teacher victimization have received less attention. Understanding how to reduce teacher-directed violence is vital to preserving the overall well-being of teachers, supporting their teaching, reducing teacher burnout, and providing recommendations for future research. The current scoping review reports on teacher-directed violence interventions in K–12 school settings (*n* = 2). In addition, emerging evidence is described that provides recommendations for developing teacher-directed violence interventions (*n* = 6). The results from this scoping review reinforce the need for further study of teacher victimization-focused interventions to support the essential work of teachers.

## 1. Introduction

School-based violence is a pervasive issue, and one rising manifestation of school-based violence includes violence against teachers (Longobardi et al., 2019). Recent reports have indicated that this type of violence manifests in various forms and occurs at high rates worldwide (Bare et al., 2021; Berlanda et al., 2019; McMahon et al., 2024; Reddy et al., 2018). Over the past two decades, a significant body of literature has been dedicated to comprehending the ordeals of educators encountering violence in the context of their jobs. Most of this research has concentrated on unveiling the extent of this form of violence and aggression (Wilson et al., 2011). While this issue is widespread, evidence-based methods to mitigate the issue remain understudied (McMahon et al., 2020a).

### 1.1. Prevalence and Correlates of Teacher-Directed Violence: A Global Issue

A growing body of literature has shown that the issue of violence against teachers is widespread and transcends geographical boundaries, affecting educators on a profound scale. In a meta-analysis that included 24 studies, Longobardi et al. (2019) found that physical (e.g., attacks) and non-physical (e.g., intimidation) teacher-directed violence ranged from 20% to 75%, with a pooled prevalence of 53% within the previous two years. A recent study in the United States led by McMahon et al. (2024), utilizing a sample of 11,814 teachers across all 50 states, found that 80% of teachers reported verbal and threatening violence and 56% reported physical violence from students in the past year; these rates were followed by verbal and threatening and physical violence against teachers from parents (63%, 26%), colleagues (49%, 27%), and administrators (48%, 26%), respectively. Verbal aggression ranks as the most prevalent form of violence reported by teachers, followed by physical and property-related aggression (Bounds & Jenkins, 2018; McMahon et al., 2014). In Virginia (U.S.), threats against teachers accounted for 15.5% of the cases evaluated, and 5.8% of these threats resulted in attempted physical aggression (Maeng et al., 2020). The most common threats included physical aggression, such as hitting or fighting, and students with disciplinary records or receiving special education services were more likely to make these threats.

Teacher victimization has been reported worldwide, including instances of verbal and physical aggression against teachers in Jordan, as documented by Alzyoud et al. (2016), highlighting the need to strengthen training and psychological support programs for teachers. Furthermore, de Ceballos and Carvalho (2021) noted that, in Latin America, violence against teachers is a common and often underreported issue, which limits effective institutional responses and perpetuates the vulnerability of educators. Sungu (2015) highlights that the lack of clear policies and government support in several African countries exacerbates the problem, leaving it unresolved.

In Taiwan, Chen and Astor (2009) found that tensions between academic expectations and student behavior are a common source of violence, suggesting that improving the quality of interaction between teachers and students could reduce these incidents. In South Korea, Moon et al. (2015) observed high rates of violence toward teachers, which has prompted the implementation of conflict management training programs as a preventive measure. Similarly, a study in Australia (Stevenson et al., 2022) revealed that verbal aggression is the most frequent form of violence, followed by bullying and physical threats, highlighting the need for specific interventions to improve school safety.

In South Africa, de Wet (2010b) noted that violence against teachers is deeply related to social inequality, high crime, and a lack of resources. In Turkey, Ozdemir (2012) reported that verbal and physical assaults are common in secondary schools and that institutional responses are insufficient to protect educators. In the Middle East, Khoury-Kassabri’s (2012) study indicated that 15.5% of students had acted aggressively toward a teacher.

The perception of a positive school climate and clear policies are associated with less aggression, while teacher self-efficacy is not a determining factor in reducing violence. In Finland, Kauppi and Pörhölä (2012) studied the attributions that teachers make about the causes of their victimization, noting that these may be related to students, the institution, or the teacher him/herself and that these attributions influence who teachers seek support from. In South America, recent studies have provided valuable insights into teacher victimization and associated psychosocial consequences. In a comparative analysis of burnout syndrome prevalence among teachers in Argentina, Chile, Ecuador, and Peru during the COVID-19 pandemic, researchers found that critical levels of burnout were exacerbated by guilt. Teachers from Chile report the highest levels of psychological exhaustion, whereas Ecuadorian teachers show the least burnout, likely linked to stronger social support and institutional aid during the pandemic. These findings highlight the importance of supportive environments and policies tailored to regional contexts (Marsollier et al., 2024). In this study, greater institutional support and positive socio-emotional well-being were protective against negative emotions, such as stress and burnout. Fostering a supportive work climate to mitigate emotional and professional fatigue was emphasized (López-Orellana et al., 2021).

On the other hand, Mooij (2011) identified that teachers in the Netherlands who work in schools with low academic performance or who feel less integrated into society tend to experience more incidents of violence. In China, Yang et al. (2019) reported that 25.1% of teachers had experienced at least one form of victimization in the last school year, with social and verbal aggression being the most common. Perceived high levels of bullying at school and punitive disciplinary practices were associated with higher victimization.

### 1.2. Offenders, Characteristics, and Effects of Teacher-Directed Violence

Given the widespread nature of violence directed against teachers, researchers have started delving into the nuances of teachers’ experiences. Teachers encounter violence from diverse offenders, including students, parents, and even colleagues and administrators (de Wet, 2010a; May et al., 2010; McMahon et al., 2024). Students consistently emerge as the primary instigators of violence against teachers, with teachers reporting aggression from male students more often than from female students (Gerberich et al., 2011; Kauppi & Pörhölä, 2012). While elementary teachers report higher rates of physical aggression, middle and high school educators are more susceptible to student aggression overall (Chen & Astor, 2009; Gerberich et al., 2011; McMahon et al., 2022).

Understanding the elements contributing to teacher-directed violence is essential in devising preventive and management strategies. Risk factors include working in urban school settings and prior exposure to incidents of teacher-directed violence (Bounds & Jenkins, 2018; Melanda et al., 2019). An exploration of demographic factors reveals mixed findings. Some studies associate more work experience with lower violence rates, while others identify it as a risk factor (Bounds & Jenkins, 2018; Casteel et al., 2007; Martinez et al., 2016; Varela et al., 2021). Similarly, some studies indicate that female teachers are more likely to experience violence, while others suggest that male teachers are more at risk (Casteel et al., 2007; McMahon et al., 2014; Moon & McCluskey, 2016; Msuya, 2016; Schofield et al., 2019). Additional contributing factors include excessive workload, student performance, discipline enforcement, conflicts, and perceptions of teacher unfairness (Alzyoud et al., 2016; Chen & Astor, 2009; McMahon et al., 2020a). School environment also plays a role, with factors like school climate, discipline policies, teacher stress, and time constraints influencing violence (Khoury-Kassabri, 2012; Prpić, 2019; Steffgen & Ewen, 2007).

Violence against teachers has long-lasting physical and emotional effects on teachers and others within educational settings (Wilson et al., 2011). Violence also affects the school environment, social climate, and students’ academic performance. The repercussions of teacher-directed violence are far-reaching, encompassing personal and organizational outcomes, such as anxiety, depression, PTSD, sleep disturbances, physical health issues, strained relationships, diminished professional capacities, and increased teacher turnover (de Vos & Kirsten, 2015; Ozkilic & Kartal, 2012; Peist et al., 2020; Wilson et al., 2011). Longobardi et al. (2019) emphasize the prolonged emotional and psychological effects on teachers who are victims of violence, including lower job satisfaction. more emotional exhaustion, and intentions to leave teaching (Longobardi et al., 2019; McMahon et al., 2023). Similarly, McMahon et al. (2020b) emphasize that violence against teachers contributes to the creation of a hostile school environment, negatively affecting teaching–learning dynamics and weakening cohesion between students and educators. In addition, Wilson et al. (2011) demonstrate that the insecurity perceived by teachers can lead to higher rates of absenteeism and an intention to leave the profession.

### 1.3. School-Based Interventions

This escalating and pervasive concern necessitates immediate attention. Violence against teachers encompasses a wide range of forms, from physical and verbal aggression to relational harassment, sexual harassment, and property damage. These manifestations of violence, while varying in frequency and context depending on the setting, reflect an urgent problem that requires effective measures tailored to the circumstances of each educational community.

One of the most widely adopted strategies to address this problem is school-based interventions designed to prevent or address school violence by creating a safe and supportive school environment. These interventions range from conflict management training programs to restorative discipline policies that reduce recidivism and encourage peaceful dispute resolution (Carroll et al., 2022; Perry et al., 2024). In countries such as the United States, these interventions have been implemented in response to the increasing number of incidents, with initiatives such as monitoring incidents of violence, emotional management training for students and teachers, and the introduction of school-based counselors to address risk factors related to violent behavior (Espelage et al., 2013; McMahon et al., 2020a). Maeng et al. (2020) indicated that school responses to physical threats in the U.S. included suspensions, changes in placement, and, in some cases, mental health interventions.

Countries worldwide have addressed violence against teachers with approaches tailored to their cultural and socioeconomic contexts. For example, in South Korea, facing high rates of violence against teachers, conflict management training programs targeting both students and teachers were implemented. This included strengthening communication and building mutually respectful relationships to reduce violent classroom incidents (Moon et al., 2015). In Australia, for instance, in response to the prevalence of verbal aggression and threats, the Australian government promoted intervention policies ranging from the presence of counselors in schools to the implementation of clear protocols for handling incidents of violence (Stevenson et al., 2022). These measures seek to improve school safety and provide psychological support to affected teachers and students.

Violence in South African schools is closely linked to socioeconomic factors, such as social inequality and high crime rates. In this context, the government has encouraged community involvement in schools and has implemented positive discipline policies that promote collaboration between families and educators to reduce violence (de Wet, 2010b).

In Turkey, the frequency of verbal and physical aggression in secondary schools led to the implementation of awareness campaigns on the importance of respect for teachers and the development of protocols for reporting incidents and ensuring effective institutional responses (Ozdemir, 2012).

On the other hand, in Finland, intervention has focused on training teachers to identify and manage risk factors associated with victimization, such as the perception of bullying in the school environment. In addition, psychological support has become an essential strategy for teachers who have been victims of aggression, fostering resilience and emotional stability (Kauppi & Pörhölä, 2012). Finally, in response to high levels of verbal and physical aggression against teachers, the Jordanian government has implemented psychological support and training programs for handling violent situations to reduce the incidence of these events and improve the well-being of teachers (Alzyoud et al., 2016).

### 1.4. Need for Standardization in Evaluation

Although numerous programs and initiatives are designed to mitigate school-based violence, we have a lack of standardization in the evaluation methods and analysis tools used to measure their effectiveness, particularly for violence against teachers. The diversity of approaches makes it difficult to compare and consolidate the data to achieve a holistic understanding of the most effective interventions. Researchers have emphasized the need to develop standardized evaluation tools that allow comparisons of outcomes and provide clear guidance for future interventions (Reddy et al., 2018). Teacher safety may also help motivate teachers to be involved in efforts to improve school safety and violence prevention, as they are often the front line for implementing school safety measures. Using a scoping review, we seek to consolidate the current knowledge on interventions in different contexts, providing a basis for future policy and research to protect teachers and improve the overall educational environment by understanding current interventions designed to prevent or mitigate teacher victimization.

## 2. Materials and Methods

### 2.1. Search Strategy

We conducted a scoping review in accordance with JBI methodology guidelines and reviewed the empirical literature focused on the victimization of school personnel (See Appendix A). We used a protocol informed by the Preferred Reporting Items for Systematic Reviews and Meta-Analysis (PRISMA) guidelines to search bibliographic databases, screen articles, apply inclusion and exclusion criteria, and select the relevant literature for this scoping review (Moher et al., 2009). With the help of a trained librarian, we developed a comprehensive search to identify publications using PsycInfo, ERIC, Medline, and Social Science Abstracts.

We restricted our search to English-only articles and collected all database results published in the years between 1990 and 2024. We used terms such as “teacher” OR “educator” OR “school personnel” AND “violence” OR “aggression” AND “intervention” OR “curriculum” OR “policy” as a basis for our key search constructs (see Appendix B for specific search terms and syntax). Subsequently, the text words in the titles and abstracts of relevant articles, along with the index terms describing the articles, were employed to develop criteria for determining our final review sample.

### 2.2. Inclusion and Exclusion Criteria

Articles reporting on interventions for school personnel’s experiences of victimization within a K–12 school setting were included in this review. Articles available in English were eligible for inclusion. We excluded articles that (1) did not include or describe interventions to prevent school personnel’s victimization; (2) were not conducted in a K–12 school setting; (3) were not published in English; and/or (4) were a review, editorial, opinion piece commentary, dissertation, or solely reported on implications for practice.

### 2.3. Study Selection

**Figure 1:**
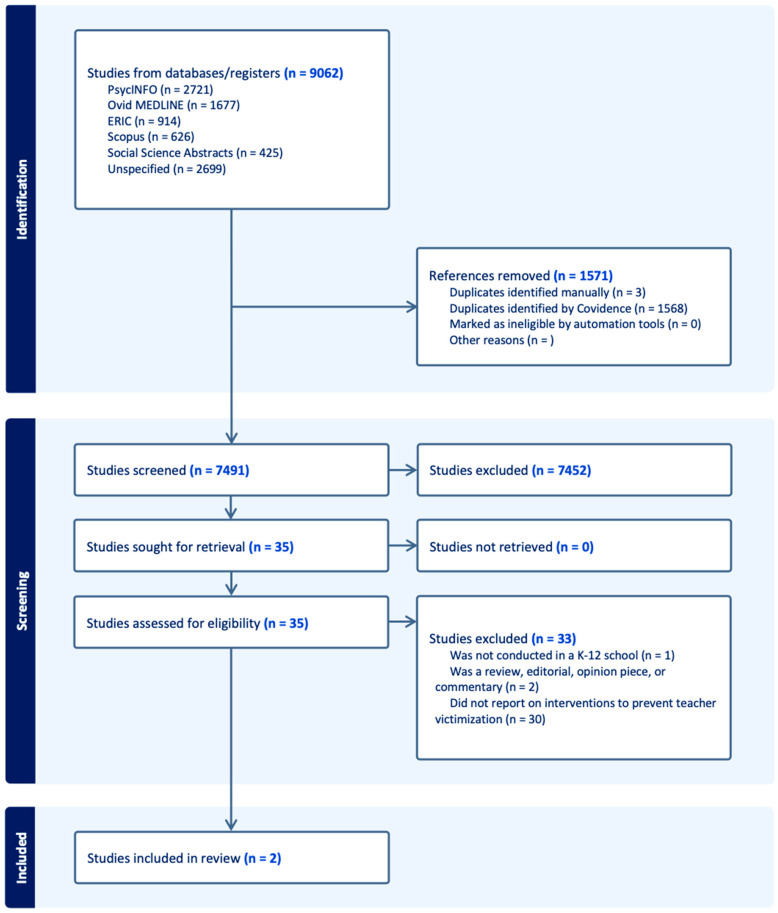
Scoping review search process.

Using Covidence web-based review software, a title and abstract review was conducted. Two reviewers blindly and independently screened each title and abstract for the inclusion/exclusion criteria. The full study team met to resolve discrepancies. The team followed a similar procedure for the full-text review, whereby two reviewers blindly and independently reviewed the full text of each article that passed the title/abstract screening, and the study team met to resolve disagreements. All excluded studies were documented with the reasons for their exclusion in a flow chart, in adherence to the PRISMA flowchart (Figure 1).

### 2.4. Data Extraction

Following the full-text review, the reviewers extracted data from the full text of the articles that met the inclusion criteria. For this study, we extracted study characteristics, including author and publication year, setting (i.e., urban, rural, suburban), school type, school/student characteristics, geographic location, sample size, and gender, race/ethnicity, and years of teaching experience of school personnel. We also extracted information regarding study design (i.e., observational, quasi-experimental, and experimental), theory guiding the work, control variables, whether it was in-service training, outcome measured, length of intervention, type of victimization, victimization characteristics, and evaluation results. A member of the study team performed the initial extraction of information, which was then reviewed for quality and accuracy by a second reviewer. The team resolved discrepancies through discussion and consensus.

## 3. Results

The search strategy yielded a total of 9062 articles (Figure 1). Our initial database search identified 7491 unique articles. During our initial title/abstract screening, we excluded 7452 articles based on the four criteria described above (i.e., not intervention, K–12 schools, in English, or commentary). Among the 35 articles remaining, two studies met our full inclusion criteria and were included in the final sample. The studies excluded were based on the following: not conducted in K–12 schools (*n* = 1), was a review or editorial (*n* = 2), or did not report on an intervention designed to prevent or reduce violence against teachers (*n* = 30). During the screening process, the review team recognized several articles that were categorized as “emerging evidence.” These studies provide examples of different types of school practices and strategies that inform interventions. These studies are reported separately, categorized as “emerging evidence,” and reported in Table 1 and Table 2, respectively.

The following section presents the results, organized by individual study. The results report authors and year, study design, and location and school type where the intervention was implemented. A description of the intervention is provided, followed by the characteristics of the teachers who engaged in the intervention and the description of victimization perpetrated against the teachers). Each study also includes an overall description of the outcome, depicting what the study accomplished, and future directions posited by the authors. Studies are organized by those that met overall inclusion (N = 2), followed by a summarized section that includes emerging evidence (N = 6).

### 3.1. Studies Included in the Review

#### 3.1.1. Study 1—Leuschner et al. (2017)

This study investigated the NETWASS Prevention Model for Targeted School Violence and found that the intervention did not influence feelings of safety for school staff but did for the crisis prevention team (CPT), which includes specially trained members of the school staff, the school’s principal, teachers, and social workers. They conducted a quasi-experimental study of the NETWASS Prevention Model for Targeted School Violence in 98 public and private schools (n = 3473 school staff members) in Germany to evaluate teachers’ knowledge about school shootings, their sensitivity to warning signs of violent behavior, and their ability to assess crisis symptoms and handle psychosocial crises adequately. The NETWASS Prevention Model for Targeted School Violence is a threat assessment model that trains teachers on how to recognize behaviors that indicate psychosocial crises in children that could lead to violent behavior, assess the behaviors, and apply the appropriate measures to mitigate risk.

The evaluation design included three different implementation conditions: (1) the “Extensive condition” (32 schools): two-day training in crisis prevention for a CPT (3–12 people) composed of teachers, social workers, and administrators, followed by a two-hour training with other school staff, with both trainings being conducted by psychologists of the NETWASS research team of Freie University; (2) the “Multiplier condition” (37 schools): two-day training in crisis prevention for the CPT conducted either by a school psychologist or by police officers who had received a specific NETWASS multiplier training and a two-hour training with other school staff conducted by a school principal or a member of the CPT; and (3) the “Self-instruction condition” (29 schools): 2 h of presentation of an information booklet for the CPT and school staff, at the same time, without separate or specific training. The researchers examined in-person threats of school violence and reported that teachers have mixed feelings regarding perceptions of safety. The authors found that the intervention had a positive effect on feelings of safety for CPT members (who received the two-day training) but not for school staff (who received the two-hour training; d^0^STAFF = −0.116; d^0^CPT = −0.158) between the baseline and follow-up.

Other differences were found in school-shooting expertise (SSE), evaluation skills (ES), and evaluation certainty (EC) for school staff (STAFF) and crisis prevention teams (CPTs). Across all conditions, significant improvements were observed in the SSE between baseline and post-intervention (βextensive = 0.374, SE = 0.063, *p* < 0.001; βmultiplier = 0.400, SE = 0.093, *p* < 0.001; βself = 0.313, SE = 0.080, *p* < 0.001). Effect sizes (d’) were consistent across conditions, ranging from 0.184 to 0.221. Changes in SSE between post-intervention and follow-up were negative but not statistically significant in the self-instruction condition. Improvements in ES were significant across all conditions from baseline to post-intervention (βextensive = 2.504, SE = 0.263, *p* < 0.001; βmultiplier = 1.625, SE = 0.270, *p* < 0.001; βself = 2.003, SE = 0.423, *p* < 0.001), with the CPT showing greater benefits in the multiplier condition (β = 1.215, SE = 0.555, *p* < 0.05). Reductions in ES between post-intervention and follow-up were significant for the extensive (β = −1.245, SE = 0.313, *p* < 0.001) and self-instruction (β = −1.001, SE = 0.418, *p* < 0.05) conditions. EC improved significantly for all conditions between baseline and post-intervention (βextensive = 2.896, SE = 0.306, *p* < 0.001; βmultiplier = 2.133, SE = 0.768, *p* < 0.01; βself = 2.898, SE = 0.664, *p* < 0.001), with the CPT showing less benefit in the self-instruction condition compared to STAFF. Overall, Leuschner et al. (2017) reported better outcomes for the first two conditions compared to the last condition. They did not report on the differences between conditions for feelings of safety, trust in organizational structures, teacher–student interaction, or school staff cohesiveness.

#### 3.1.2. Study 2—Stelko-Pereira and Williams (2015)

This study examined a Brazilian school violence prevention program (Violência Nota Zero) and found improved teacher mental health and a reduction in student engagement in violence. They conducted a delayed intervention experimental study to test their intervention, Violência Nota Zero, in two public schools in Sao Paulo, Brazil. The control and intervention schools were located within six blocks of each other in a region with a low socioeconomic status, a lower level of education, and a large concentration of young families. The authors implemented a 12-week series of 90 min face-to-face meetings and activities during the week with teachers and school counselors. The intervention sought to reduce violence and bullying in schools between students and among staff and students. Activities included a presentation on punishment in schools, child abuse, and maltreatment and a discussion of strategies to deal with crisis situations. Researchers conducted a pre-and post-program evaluation and eight-month follow-up at the intervention school. The authors surveyed seven teachers (all female) and one counselor from the intervention school and six teachers and one counselor (five female, two male) from the control school, for a total of 15 educators.

The authors conducted a comparison of the two schools using the Wilcoxon test and found that teachers in the intervention school reported improved mental health (z = −2.1, *p* = 0.03 compared to z = −1.86, *p* = 0.06). Comparing pre-intervention and follow-up after 8 months of intervention at the treatment school, the researchers reported a reduction in educator victimization by students (z = −1.2, *p* = 0.20 compared to z = −1.90, *p* = 0.04). Stelko-Pereira and Williams (2015) found no differences in teacher victimization by students between the intervention and control schools when comparing the pre- and post-program data for both schools.

### 3.2. Emerging Evidence Informing Intervention Development

Six studies are described below that examine policies or practices that may inform the development of future teacher-directed violence interventions. For example, Bass et al. (2016) investigated how exposure to student violence affects burnout and engagement levels among school staff, including teachers, counselors, and support staff within a large school district in the Northeastern United States. In this study, 728 staff members completed an anonymous survey that measured burnout with the Maslach Burnout Inventory, engagement with the Utrecht Work Engagement Scale, and experiences of various forms of student violence, such as verbal insults, physical aggression, threats, and sexual harassment. Additionally, the survey assessed employees’ perceptions of safety within their schools and their leaders’ transformational qualities, including supportiveness and inclusivity. Using structural equation modeling, Bass et al. (2016) found that higher exposure to student violence strongly predicted increased burnout (β = 0.52, *p* < 0.001) and decreased engagement (β = −0.45, *p* < 0.001). The analysis also identified a mediating effect of perceived school safety; employees who felt less safe at school due to violence reported higher burnout and lower engagement. Transformational leadership buffered these negative effects, with employees reporting lower burnout and higher engagement when they perceived their leaders as supportive and encouraging, even in high-violence environments (interaction term for transformational leadership on engagement, β = −0.30, *p* < 0.01). The study emphasized the importance of transformational leadership in fostering resilience among school staff that face challenging environments.

Kapa et al. (2018) conducted a large-scale quantitative study using data from the 2011–2012 Schools and Staffing Survey (SASS) to explore the effect of authoritative school environments on teacher victimization rates. Their sample included 37,497 teachers across 7844 U.S. public schools, where they examined experiences of verbal threats and physical attacks by students. Kapa et al. (2018) assessed “structure” within schools as consistent rule enforcement by principals and teachers, while “support” was defined by indicators like parental involvement and student behavior, including reduced tardiness and absenteeism. Through hierarchical generalized linear modeling (HGLM), the study found that schools with consistent rule enforcement by principals had significantly reduced odds of teachers experiencing threats (β = −0.073, *p* < 0.01) and physical attacks (β = −0.092, *p* < 0.01). Similarly, rule consistency among teachers contributed to a lower risk of victimization (β = −0.044, *p* < 0.01 for threats; β = −0.065, *p* < 0.01 for physical attacks). Supportive environments with fewer student behavioral issues also offered protective benefits, reducing teacher victimization rates. High levels of parental involvement and administrative support were associated with lower odds of victimization, while schools with frequent tardiness, absenteeism, and low parental engagement saw increased victimization. The findings suggest that fostering authoritative environments with both structure and support may enhance school climate and reduce the factors contributing to teacher stress, victimization, and absenteeism.

McCluskey et al. (2024) explored the influence of procedural and distributive justice on teachers’ satisfaction with administrative responses to victimization incidents. The study’s sample included 4005 middle and high school teachers across the 50 largest school districts in the United States, specifically focusing on theft, physical assault, and sexual harassment incidents. McCluskey et al. investigated how perceptions of procedural justice (e.g., fair treatment, respect, and active listening) and distributive justice (e.g., disciplinary actions like detention or suspension) affected teacher satisfaction. The study employed ordinal regression analysis and found that procedural justice significantly predicted teacher satisfaction (b = 2.19, *p* < 0.001), with increased perceptions of fairness associated with a higher likelihood of satisfaction (factor increase of 8.97). Distributive justice, specifically through severe disciplinary actions, also correlated with higher satisfaction levels (OR = 9.02). Apologies from offending students were associated with increased satisfaction but became less significant when procedural justice was included in the model, suggesting procedural justice as a more robust predictor. These results highlight that fair treatment and appropriate disciplinary measures can substantially improve teacher satisfaction with administrative responses to victimization incidents.

Moon et al. (2021) addressed teacher satisfaction with school responses to victimization events, focusing on how different administrative actions influenced satisfaction levels. The study surveyed 1628 teachers in an urban school district in the Southwestern United States, with the sample primarily comprising female teachers (71%) and those identifying as Hispanic (42%) or White (50%). The teachers reported experiencing theft, sexual harassment, and physical assault and assessed their satisfaction with administrative responses, such as questioning students, assigning detentions, suspensions, or taking no action. The results, analyzed via ordinal logistic regression, showed that over 50% of victimized teachers were dissatisfied with administrative responses. Satisfaction increased significantly when administrators questioned or disciplined students, with higher odds of satisfaction reported for questioning (OR = 2.23, *p* < 0.05) and suspensions (OR = 3.57, *p* < 0.01), compared to no action. Procedural justice also predicted satisfaction, as teachers who felt administrators applied procedures fairly were more likely to be satisfied (b = 2.19, *p* < 0.001), increasing satisfaction odds by 8.97. The study underscores the importance of appropriate administrative actions and procedural justice in fostering teacher satisfaction following victimization.

Perry et al. (2024) conducted a study on teachers’ perceptions of the effectiveness of school-based interventions against violence in pre-kindergarten to 12th-grade settings. Drawing from a sample of 4471 teachers, the study analyzed four intervention types: exclusionary discipline (e.g., suspensions), school hardening (e.g., metal detectors), prevention (e.g., social–emotional learning), and crisis intervention (e.g., de-escalation techniques). The results showed that prevention practices were rated as the most effective in reducing violence (mean effectiveness = 3.67, SD = 0.85). School-hardening practices were negatively associated with verbal and physical violence (verbal OR = 0.83, *p* < 0.001; physical OR = 0.78, *p* < 0.001). In contrast, exclusionary discipline and crisis intervention practices were linked to higher risks of violence, with crisis interventions positively associated with physical violence (OR = 1.12, *p* < 0.05). These findings suggest the need for schools to prioritize preventive measures and appropriate disciplinary practices to improve safety and reduce violence against educators.

Finally, Payne and Gottfredson (2019) examined how communal school organization—characterized by cohesion and a sense of community—affects teacher victimization. Using a national sample of 37,497 teachers, they explored experiences of verbal threats and physical attacks within a supportive school environment. The results indicate that schools with high levels of communal organization report lower odds of teacher threats (OR = 0.727) and physical attacks (OR = 0.818). Additional factors, such as school size and urban location, increase the likelihood of victimization, with teachers in larger or urban schools facing greater risks of both threats and physical violence. Teachers working in special education were particularly vulnerable to victimization. Overall, the study highlights the protective role of communal organization in reducing violence toward teachers and underscores the importance of fostering positive, cohesive school environments.

## 4. Discussion

Our review of interventions addressing teacher victimization in schools reveals a notable gap in the research. Although the prevalence of educator-directed violence is well-documented globally, the results of our search and analysis suggest that intervention research is still in its early stages, with only two studies meeting the full inclusion criteria. This finding is especially concerning given that Longobardi et al. (2019) estimate that between 20 and 75% of teachers experience victimization at some point in their careers, whether through direct threats, physical aggression, relational harassment, or systemic issues, such as exposure to community violence and the threat of school shootings. Our results underscore the urgent need to consider teacher perspectives, safety, and experiences when developing programs and policies to improve school safety and prevent violence.

The two intervention studies reviewed, Leuschner et al. (2017) and Stelko-Pereira and Williams (2015), yielded mixed results, highlighting both the potential and limitations of current approaches. Leuschner et al. (2017) found that, while the NETWASS Prevention Model improved school staff’s crisis assessment skills, it had minimal impact on teachers’ perceptions of safety, suggesting that crisis prevention training may not directly address broader issues of teacher victimization. Similarly, Stelko-Pereira and Williams (2015) found improvements in teacher mental health and a reduction in student violence, but no significant changes in teacher victimization. Our review highlights that intervention studies on teacher victimization are limited, and the two studies included in this review exhibit several methodological weaknesses. While both studies used a control group design, which is a strength, one of the studies had a very small sample size of only 15 participants, limiting the generalizability of its findings. Moreover, the focus of these outcome studies was not on actual teacher victimization, but rather on perceptions of safety, trust in organizational structures, teacher–student interactions, and school staff cohesiveness, as well as the potential antecedents of victimization. Although changes in attitudes may be important and could relate to behavior, the primary goal of violence prevention should be to reduce teacher victimization from all forms of violence. Additionally, the reviewed studies lacked clear conceptual frameworks guiding the interventions. This is a notable concern, as conceptual models are crucial for identifying mechanisms of change and mediating variables, enabling replication across different settings and populations, and providing a structured approach for advancing evidence-based strategies in the field.

The emerging body of research we included in our review highlights several key themes that can inform effective strategies for reducing teacher victimization. One critical factor identified across multiple studies is the role of school leadership and the cultivation of a positive school climate. Bass et al. (2016) found that transformational leadership, which promotes a supportive and inclusive school culture, significantly mitigated the negative impact of student violence on teacher well-being. Similarly, Kapa et al. (2018) emphasized that consistent rule enforcement and a supportive environment are crucial for reducing teacher victimization. These studies suggest that fostering positive school climates and empowering leadership are essential strategies for addressing the underlying causes of teacher victimization.

Another theme that emerged is the importance of procedural and distributive justice in improving teacher satisfaction with administrative responses to violence. McCluskey et al. (2024) and Moon et al. (2021) found that teachers who perceived fair treatment and appropriate disciplinary actions were more satisfied with how their victimization was handled. These findings underscore the need for administrative responses that are not only effective but also perceived as fair, with a focus on transparency and accountability. Ensuring fairness in the handling of victimization can help reduce teachers’ feelings of vulnerability and improve their overall sense of safety and job satisfaction.

In addition to leadership and justice, specific approaches to school violence prevention and broader environmental factors also play a significant role in promoting teacher safety. Perry et al. (2024) demonstrated that preventive practices, such as social–emotional learning programs, are more effective at reducing violence than punitive measures like suspensions. Likewise, Payne and Gottfredson (2019) identified a strong communal school organization as a protective factor against teacher victimization. These studies suggest that a holistic approach to intervention—one that promotes not only individual behavior change but also a cohesive and supportive school culture through a trauma-informed lens—can be more effective in reducing teacher victimization.

Finally, the evidence highlights the importance of context-specific interventions that account for the unique challenges faced by different schools. McCluskey et al. (2024) and Perry et al. (2024) both emphasized that teachers’ perceptions of the effectiveness of school interventions are influenced by the type of violence they experience and how well interventions are tailored to their specific needs. Additionally, Payne and Gottfredson (2019) pointed out that school characteristics, such as size and location, significantly impact victimization rates, indicating that interventions must be adapted to the specific context of each school. These insights suggest that tailored, context-specific strategies, designed with educators’ perspectives in mind, are essential for creating effective violence reduction programs.

Most interventions focused on school violence prevention are student-centric, often overlooking the victimization of teachers. Park-Higgerson et al. (2008), for example, identified 26 randomized control trials for school-based violence prevention but found few that addressed teacher experiences directly. Lester et al. (2017) observed a similar trend, noting an absence of reviews focused on student-to-teacher violence. These reviews primarily examined peer aggression or intimate partner violence among students, with minimal attention given to violence directed against school personnel. Though necessary for student safety, this focus inadvertently neglects teachers, whose victimization can have cascading effects on the entire school ecosystem. In addition, problematic teacher behaviors toward students, parents, and colleagues should also be assessed. Our findings suggest that school safety interventions need to recognize the unique victimization experiences of teachers and broaden their scope to include all members of the school community.

Teacher-focused interventions have implications for whole-school environments and school safety because their involvement and leadership are vital for the success of efforts to create safe school environments. Lateral surveillance and reporting technologies, including Mental Health First Aid (Kelly et al., 2011) and anonymous reporting systems are predicated on building trust and positive relationships across everyone in the school community. Bullying and a lack of support from administrators experienced by teachers may interfere with the effective implementation of other types of school violence prevention programming, given that teachers are often involved in implementing or enforcing such programs (de Wet, 2010b; McMahon et al., 2017). Reducing the violence burden borne by teachers will contribute to their health and well-being and, by extension, the effectiveness and sustainability of school safety programs more generally because they are often involved in creating a positive school climate, implementing programs and policies for school safety, and role-modeling appropriate behavior for the student. In other words, school violence prevention requires all members of the school environment to feel and be safe.

### Future Directions

Although there is growing awareness of the problem of teacher victimization, much more work remains to be conducted to develop comprehensive, evidence-based interventions. Future research should focus on refining intervention strategies, using rigorous research designs, and exploring long-term outcomes to better support teachers and create safe, conducive learning environments for all school stakeholders. Furthermore, interventions should be designed to address both the immediate and systemic causes of teacher victimization, considering factors such as school leadership, climate, and community support. Examining previous reviews on school violence, Lester et al. (2017) also found no reviews centered on student violence against teachers. Rather, most researchers focused on peer aggression and intimate partner violence in student populations. Moreover, we found only one example of emerging evidence that expanded the measurement of teacher victimization beyond student aggressors to include parents and colleagues (e.g., sexual harassment, bullying). This suggests that school safety and violence prevention may also need to include attention to parent–teacher interactions and education among teachers and other school personnel about appropriate behavior among colleagues and supervisors.

Our results suggest several actions that may help move the field to a more comprehensive approach to school safety that considers teachers in efforts to make our schools safer (Stilwell et al., 2024). These strategies need to be designed to include attention to teacher victimization prevention and aftercare. Yet, in considering more strategies that also include teacher-directed violence, our results suggest several issues may require attention.

First, teacher victimization interventions need to be theoretically driven. This is necessary because it provides a conceptual framework that can be replicated and focuses on the factors that are known to be correlated with reductions in victimization or assisting with coping with the experience. Theory will also help identify school-based factors that facilitate or protect against violence and may inform how the intervention may need to be tailored to the range of potential perpetrators of teacher violence. A conceptual framework will also help inform the outcome measures that are most appropriate for a particular intervention. Building interventions grounded in theory and considering school, neighborhood, region, and cultural context also helps focus the intervention, as it is unrealistic to expect one intervention to address the complex and multifaceted nature of teacher victimization.

Second, the evaluation methods applied need to ensure confidence in the external and internal validity of the study. This means studies need to include large representative samples of teachers or schools to increase the likelihood that the results generalize to similar contexts. While no one study can be completely representative of all teachers, researchers need to consider the characteristics of teachers and schools with large enough samples to generalize in some way. It is possible, for example, that teachers in urban schools may have different victimization experiences than those in more rural schools. Non-white teachers in predominantly white schools may have different experiences than teachers in schools with student bodies that are ethnically like themselves. Male and female teachers may have different experiences and respond to interventions differently. It is also possible that elementary teachers may differ in important ways from middle school or high school teachers, which likely requires further tailoring of prevention strategies. It is vital that the sampling for the intervention studies includes large enough samples so that efforts to explore how teacher and contextual characteristics might affect outcomes may be adequately powered.

Rigorously designed research is also necessary if we are to gain confidence in the internal validity of intervention trials. Random assignment to an intervention or control condition is considered the most ideal design, but this may not be feasible for practical and ethical reasons. Nevertheless, longitudinal mixed-method designs that include comparison and control conditions are necessary, if we are to reduce alternative explanations of the intervention and establish confidence in the evidence base for it. Designs also need to consider school-level effects because different school cultures may influence the implementation or effectiveness of an intervention. As the field develops, implementation studies and in-depth qualitative analysis about implementation are also appropriate, but the field is so limited that establishing an evidence base of effective interventions is necessary before—or, ideally, while—considering these kinds of questions.

Third, efforts to address teacher victimization might benefit from greater integration into existing school-based practices. We found threat assessment, for example, used in one study (Leuschner et al., 2017) that also included teachers as victims. This is a promising approach because threat assessment is implemented broadly in U.S. schools. This strategy could also involve administrators, counselors, and social workers who can help support teachers (Leuschner et al., 2017; Stelko-Pereira & Williams, 2015) who also experience violence. Interventions, including trauma-informed practices, should be broadened to include all school stakeholders, including students, teachers, administrators, and staff (McMahon et al., 2024).

Finally, our review suggests that more development of standardized measures would facilitate an understanding of the ecology of schools, the conditions that lead to violence, and effective policies and practices that prevent and reduce violence against teachers and students. Standardized measures and annual assessments will facilitate comparisons across schools, geographical contexts, and time periods, including during the transition to post-COVID-19 conditions. This approach will help identify trends, evaluate the effectiveness of interventions, and guide the development of policies that seek to prevent violence in diverse settings. We can learn from schools in high-risk communities that are succeeding and have low rates of student–student and student–teacher violence. We need a holistic approach that is tailored to the school context and enables assessment of what strategies work best in specific contexts.

In sum, educator-directed violence is a widespread problem, and research has grown significantly. Yet, there are very few intervention studies to date, and these studies have notable methodological challenges. There is an urgent need to go beyond prevalence to develop and evaluate theory-driven, ecologically informed interventions using rigorous designs, trauma-informed methods, and valid and reliable measures to effectively address, reduce, and prevent school violence for teachers and all school stakeholders.

## Figures and Tables

**Table 1:** Intervention characteristics, intervention protocols, and descriptions (*n* = 2).

Author (Year)	Location/ Setting/Sample Size	School Type	Intervention Description	Teacher Characteristics	Victimization Characteristics	Intervention Outcome
Leuschner et al. (2017)	International/Berlin, Brandenburg, and Baden–Wurttemberg (n = 3473)	Private and Public	Crisis Prevention Team (2 days training for some and 2 h for others and 7 months follow-up) consisting of 3 to 12 people for all school staff	66.8% female	In-person: threats of school violence	Mixed feelings regarding safety of teachers.
Stelko-Pereira and Williams (2015)	International/ Sao Paulo, Brazil (n = 15)	Public	12 weekly meetings of 90 min with teachers and school counselors	School A: 8 teacher participants females. School B: 5 females, and 2 were males.	In-person: mental health of teacher and victimization experience	Improvements in teachers’ mental health problems were noticed after the intervention, in comparison to the control school

**Table 2:** Emerging evidence, study protocols, and descriptions (*n* = 6).

Author (Year)	Location/ Setting/ Sample Size	School Type	Description of Effort	Outcome	Teacher Characteristics	Victimization Characteristics
Bass et al. (2016)	US (n = 728)	Public	Association between school staff victimization and burnout/work engagement with potential mediation effects of staff perception of school safety and potential moderation effect of staff perception of school leadership	School staff victimization was significantly, positively associated with burnout and significantly, negatively associated with work engagement. Perception of school to be unsafe mediated the relationship between school staff victimization and burnout/work engagement. Transformational school leadership moderated the association between staff victimization and perception of school to be unsafe. It did not moderate the association between staff victimization and burnout/work engagement.	Female (80%) White (76%) Teachers (57%) Other Staff (43%)	In-person (insults, threats, physical assault, sexual harassment)
Kapa et al. (2018)	US (n = 37.49)	Public	Effect of authoritative discipline on teacher victimization	Schools where teachers state that administration enforces school rules on student conduct had a significant reduction in teacher victimization compared to schools where teachers did not perceive administration to enforce rules.	Female 69%	In-person (verbal threat, physical assault)
McCluskey et al. (2024)	US/multi-state (n = 4005)	Public	Teacher satisfaction with school administration’s handling of victimization events Procedural justice: admin treated with respect, treated fairly, took time to listen, based decisions upon fact, explained decisions, and intended to respond fairly Distributive justice: doing nothing, questioning/investigating, detention, suspension/expulsion	Teachers who perceived treatment by admin to be fair were more likely to be satisfied with admin response Greater DJ (i.e., more severe response) was associated with higher levels of teacher satisfaction with admin response	Female (75%) Middle School (58%)	In-person (theft/property, sexual harassment, physical assault)
Moon et al. (2021)	US (n = 1628)	Public	Teacher satisfaction with administration response to victimization with additional predictors (type of intervention and student apology)	Higher odds of satisfaction in response when admin questioned students or disciplined students (compared to no action) >50% of teachers were dissatisfied or very dissatisfied with how admin responded	Female (71%) White (50%) Hispanic (42%) Middle and High School	In-person (theft/personal property, sexual harassment, physical assault)
Payne and Gottfredson (2019)	US (n = 37, 497)	Public	Association between level of communal school-based organization and likelihood of teacher victimization	Schools with more communal organization are less likely to report physical assault	Female (76%) Elementary and Secondary	In-person (verbal threat, physical assault)
Perry et al. (2024)	US (n = 4471)	Public	Teacher perception of effectiveness of school-based approaches to discipline	Negative association between teacher perception of school-hardening practices and teacher-directed violence Negative association between teacher perception of school prevention practices and verbal/physical violence Positive association between teacher perception of crisis intervention practices and physical violence	Female (78%) White (84%) Middle and High School	In-person (verbal, physical, property theft)

## Data Availability

The data presented in this paper are available in Table 1 and Table 2. Appendix A provides the PRISMA-ScR checklist, and Appendix B displays search terms used for the review.

## Appendix A

### Preferred Reporting Items for Systematic Reviews and Meta-Analyses Extension for Scoping Reviews (PRISMA-ScR) Checklist

**Table:**

Section	Item	PRISMA-ScR Checklist Item	Reported on Page #
**Title**			
Title	1	Identify the report as a scoping review.	1
**Abstract**			
Structured summary	2	Provide a structured summary that includes (as applicable): background, objectives, eligibility criteria, sources of evidence, charting methods, results, and conclusions that relate to the review questions and objectives.	1
**Introduction**			
Rationale	3	Describe the rationale for the review in the context of what is already known. Explain why the review questions/objectives lend themselves to a scoping review approach.	5
Objectives	4	Provide an explicit statement of the questions and objectives being addressed with reference to their key elements (e.g., population or participants, concepts, and context) or other relevant key elements used to conceptualize the review questions and/or objectives.	5
**Methods**			
Protocol and registration	5	Indicate whether a review protocol exists. State if and where it can be accessed (e.g., a Web address), and if available, provide registration information, including the registration number.	5
Eligibility criteria	6	Specify characteristics of the sources of evidence used as eligibility criteria (e.g., years considered, language, and publication status), and provide a rationale.	5
Information sources *	7	Describe all information sources in the search (e.g., databases with dates of coverage and contact with authors to identify additional sources), as well as the date the most recent search was executed.	5
Search	8	Present the full electronic search strategy for at least 1 database, including any limits used, such that it could be repeated.	Appendix A
Selection of sources of evidence †	9	State the process for selecting sources of evidence (i.e., screening and eligibility) included in the scoping review.	5
Data charting process ‡	10	Describe the methods of charting data from the included sources of evidence (e.g., calibrated forms or forms that have been tested by the team before their use, and whether data charting was done independently or in duplicate) and any processes for obtaining and confirming data from investigators.	6
Data items	11	List and define all variables for which data were sought and any assumptions and simplifications made.	6
Critical appraisal of individual sources of evidence §	12	If conducted, provide a rationale for conducting a critical appraisal of included sources of evidence; describe the methods used and how this information was used in any data synthesis (if appropriate).	--
Synthesis of results	13	Describe the methods of handling and summarizing the data that were charted.	6
**Results**			
Selection of sources of evidence	14	Give numbers of sources of evidence screened, assessed for eligibility, and included in the review, with reasons for exclusions at each stage, ideally using a flow diagram.	6
Characteristics of sources of evidence	15	For each source of evidence, present characteristics for which data were charted and provide the citations.	6–10
Critical appraisal within sources of evidence	16	If conducted, present data on critical appraisal of included sources of evidence (see item 12).	--
Results of individual sources of evidence	17	For each included source of evidence, present the relevant data that were charted that relate to the review questions and objectives.	Table 1 and Table 2
Synthesis of results	18	Summarize and/or present the charting results as they relate to the review questions and objectives.	6
**Discussion**			
Summary of evidence	19	Summarize the main results (including an overview of concepts, themes, and types of evidence available), link to the review questions and objectives, and consider the relevance to key groups.	13
Limitations	20	Discuss the limitations of the scoping review process.	14
Conclusions	21	Provide a general interpretation of the results with respect to the review questions and objectives, as well as potential implications and/or next steps.	15
**Funding**			
Funding	22	Describe sources of funding for the included sources of evidence, as well as sources of funding for the scoping review. Describe the role of the funders of the scoping review.	16

JBI = Joanna Briggs Institute; PRISMA-ScR = Preferred Reporting Items for Systematic Reviews and Meta-Analyses extension for Scoping Reviews. * Where sources of evidence (see second footnote) are compiled from, such as bibliographic databases, social media platforms, and Web sites. † A more inclusive/heterogeneous term used to account for the different types of evidence or data sources (e.g., quantitative and/or qualitative research, expert opinion, and policy documents) that may be eligible in a scoping review as opposed to only studies. This is not to be confused with information sources (see first footnote). ‡ The frameworks by Arksey and O’Malley (6) and Levac and colleagues (7) and the JBI guidance (4, 5) refer to the process of data extraction in a scoping review as data charting. § The process of systematically examining research evidence to assess its validity, results, and relevance before using it to inform a decision. This term is used for items 12 and 19 instead of “risk of bias” (which is more applicable to systematic reviews of interventions) to include and acknowledge the various sources of evidence that may be used in a scoping review (e.g., quantitative and/or qualitative research, expert opinion, and policy document). Checklist from: Tricco et al. (2018).

## Appendix B. Search Syntax

### Social Science Abstracts (EBSCO)

DE “Elementary school teachers” OR DE “High school teachers” OR DE “Public school teachers” OR DE “Educators” OR DE “Teachers” OR DE “School principals” OR TI(teacher* OR educator*) OR AB (teacher* OR educator*) OR ((TI(school* OR K-12 OR “Junior High”) OR AB(school* OR K-12 OR “Junior High”)) N3 (TI(Personnel OR staff OR coach* OR administrator* OR counselor* OR “social worker*” OR psychologist*) OR AB(Personnel OR staff OR coach* OR administrator* OR counselor* OR “social worker*” OR psychologist*)))

AND

DE “Aggravated assault” OR DE “Assault & battery” OR DE “Exposure (Criminal law)” OR DE “Psychological abuse” OR DE “Rape” OR DE “Sexual aggression” OR DE “Violence” OR DE “Physical abuse” OR DE “Violence research” OR DE “Violent adolescents” OR DE “Violent children” OR DE “Youth violence” OR DE “Violent crimes” OR DE “School violence” OR DE “School bullying” OR DE “Harassment” OR DE “Harassment in schools” OR DE “Sexual harassment” OR DE “Intimidation” OR DE “Stalking” OR DE “Crime victims” OR DE “Victims” OR DE “Psychological abuse victims” OR DE “Sexual abuse victims” OR DE “Stalking victims” OR DE “Victims of violent crimes” OR DE “Victims of abuse” OR DE “Bullying” OR DE “Hostility” OR DE “Aggression (Psychology)” OR DE “Interpersonal confrontation” OR TI(violen* OR victim* OR bully* OR harass* OR assault* OR threat* OR intimidat* OR stalk*) OR AB(violen* OR victim* OR bully* OR harass* OR assault* OR threat* OR intimidat* OR stalk*)

AND

DE “Crime prevention” OR DE “Crime prevention programs” OR DE “Prevention of juvenile delinquency” OR DE “Prevention of violent crimes” OR DE “Youth participation in crime prevention” OR DE “Assertiveness training” OR DE “Diversity training programs” OR DE “Curriculum” OR DE “Education policy” OR DE “Group relations training” OR DE “Training” OR TI(“Professional development” OR train* OR prevent* OR program* OR Intervention* OR workshop* OR module* OR curriculum* OR campaign* OR Policy OR video* OR policies OR strateg* OR initiative* OR service* OR Education OR Inservice) OR AB (“Professional development” OR train* OR prevent* OR program* OR Intervention* OR workshop* OR module* OR curriculum* OR campaign* OR Policy OR video* OR policies OR strateg* OR initiative* OR service* OR Education OR Inservice)
